# Dynamics of Phyllosphere Microbiota and Chemical Parameters at Various Growth Stages and Their Contribution to Anaerobic Fermentation of *Pennisetum giganteum*

**DOI:** 10.1128/spectrum.02288-22

**Published:** 2023-04-03

**Authors:** Jie Zhao, Hao-Peng Liu, Xue-Jing Yin, Zhi-Hao Dong, Si-Ran Wang, Jun-Feng Li, Tao Shao

**Affiliations:** a Institute of Ensiling and Processing of Grass, College of Agro-Grassland Science, Nanjing Agricultural University, Nanjing, China; USDA-ARS Food Science Research Unit

**Keywords:** *Pennisetum giganteum*, growth stage, bacterial community, cooccurrence network, functional profile

## Abstract

This work evaluated the dynamic changes of phyllosphere microbiota and chemical parameters at various growth stages of Pennisetum giganteum and their effects on the bacterial community, cooccurrence networks, and functional properties during anaerobic fermentation. *P. giganteum* was collected at two growth stages (early vegetative stage [P_A]_ and late vegetative stage [P_B_]) and was naturally fermented (NP_A_ and NP_B_) for 1, 3, 7, 15, 30, and 60 days, respectively. At each time point, NP_A_ or NP_B_ was randomly sampled for the analysis of chemical composition, fermentation parameter, and microbial number. In addition, the fresh, 3-day, and 60-day NP_A_ and NP_B_ were subjected to high-throughput sequencing and Kyoto Encyclopedia of Genes and Genomes (KEGG) functional prediction analyses. Growth stage obviously affected the phyllosphere microbiota and chemical parameters of *P. giganteum*. After 60 days of fermentation, NP_B_ had a higher lactic acid concentration and ratio of lactic acid to acetic acid but a lower pH value and ammonia nitrogen concentration than NP_A_. *Weissella* and Enterobacter were dominant in 3-day NP_A_ and *Weissella* was dominant in 3-day NP_B_, while *Lactobacillus* was the most abundant genus in both 60-day NP_A_ and NP_B_. The complexity of bacterial cooccurrence networks in the phyllosphere decreased with *P. giganteum* growth. The ensiling process further decreased the complexity of bacterial networks, with the simplest bacterial correlation structures in NP_B_. There were great differences in the KEGG functional profiles of P_A_ and P_B_. Ensiling promoted the metabolism of lipid, cofactors, vitamins, energy, and amino acids but suppressed the metabolism of carbohydrates and nucleotides. Storage time had a greater influence than growth stage on bacterial community diversity, cooccurrence networks, and functional profiles of *P. giganteum* silage. Differences in bacterial diversity and functionality of *P. giganteum* silage caused by growth stage appear to be offset by long-term storage.

**IMPORTANCE** The phyllosphere microbiota consists of various and complex microbes, including bacteria with crucial relevance to the quality and safety of fermented food and feed. It initially derives from soil and becomes specific to its host after interaction with plants and climate. Bacteria associated with the phyllosphere are highly abundant and diverse, but we know little about their succession. Here, the phyllospheric microbiota structure was analyzed within the growth of *P. giganteum*. We also evaluated the effects of phyllosphere microbiota and chemical parameter changes on the anaerobic fermentation of *P. giganteum*. We observed remarkable differences in bacterial diversity, cooccurrence, and functionality of *P. giganteum* at various growth stages and storage times. The obtained results are important for understanding the fermentation mechanism and may contribute to high-efficient production without additional cost.

## INTRODUCTION

*Pennisetum giganteum* z. x. Lin, also known as Giant Juncao, is a perennial gramineous grass that is native to eastern and northeastern Africa (1). At present, *P. giganteum* has been planted in more than 80 countries and more than 30 provinces in China (2). *P. giganteum* is a C_4_ plant with high light, nitrogen, and water use efficiency and large biomass yield. It is reported that *P. giganteum* can be mowed 6 to 8 times per year and produce 254 tons of fresh grass per hectare each mowing (3). *P. giganteum* has strong resistance to waterlogging, drought, high temperature, and barren, so it is suitable for planting in humid, semihumid, arid, and semiarid regions of tropical and subtropical regions. And now, *P. giganteum* is widely used for the production of fiber products, biofuel, and livestock food (4). However, the rainy climate in planting areas and the seasonal surplus of *P. giganteum* require the development of ensiling as the preferred processing method.

Ensiling as an anaerobic fermentation process is based on the principle that available sugars in forage are fermented by lactic acid bacteria (LAB) to mainly lactic acid (5). But it should be noted that there are great differences in fermentation quality when silage is produced from forages harvested at different growth stages. Oliveira et al. (6) and Toruk et al. (7) have shown that the growth stage (or harvest date) of forage is the main factor determining the nutritional value and fermentation quality of silage. In the case of guinea grass, van Niekerk et al. (8) reported that the fermentation of guinea grass silage prepared at the early vegetative and boot stages was the lactate type, while the fermentation of silage prepared at the full bloom stage was the acetate type. These results can be closely related to the changes in the chemical composition and phyllosphere microbiota of the harvested forage. Typically, as forage grass grows, the proportions of its dry matter and cell wall components increase, while the proportions of cell contents (protein, lipids, and sugar, etc.) decrease (9). The phyllosphere microbiota, the microbes that colonize on or in the aboveground portion of plants, play critical roles in the quality and safety of silage. However, unlike the chemical composition, the distribution and succession of the phyllosphere microbiota during the growing season of *P. giganteum* and its effect on anaerobic fermentation have not been studied.

With the in-depth study of silage, the culture-based method has been unable to meet the needs of researchers to clearly understand the succession of microbial communities from forage to silage. Recent advances in culture-independent techniques, represented by high-throughput sequencing, have enabled researchers to explore the microbial community shifts involved in the forage growth and ensiling process (10). High-throughput sequencing has identified comprehensive and crucial bioinformation for an increasing number of silage studies, but the functional annotation of microbial communities is still largely unknown. Aßhauer et al. (11) reported that the characterization of phylogenetic and functional diversity is a key factor in microbial community analysis. Thus, the prediction of higher-order functional profiles underlying the silage microbial community will be highly beneficial and can be used as a supplement to 16S rRNA analysis (12).

Therefore, this work aimed to investigate the effects of growth stage and fermentation time on the fermentation quality, bacterial community, cooccurrence networks, and functional profile of *P. giganteum* silage. The obtained results may, therefore, (i) reveal the distribution and succession of the phyllosphere microbial community of *P. giganteum* and (ii) offer an in-depth understanding of the fermentative mechanism, which is of great importance for regulating anaerobic fermentation.

## RESULTS

### Phyllosphere characteristics of fresh *P. giganteum*.

The chemical and microbial compositions of fresh *P. giganteum* are shown in Table 1. Growth stage had significant (*P < *0.05) effects on all measured chemical and microbial parameters except pH value and mold count of *P. giganteum*. The crude protein (CP) content and buffering capacity (BC) decreased (*P < *0.05), while the dry matter (DM), water-soluble content (WSC), neutral detergent fiber (NDF) content, and acid detergent fiber (ADF) content and the LAB, aerobic bacterial, yeast, and enterobacterial counts increased (*P < *0.05) as *P. giganteum* grew.

**TABLE 1:** Chemical and microbial compositions of fresh *P. giganteum* harvested at two growth stages

Parameter^*a*^	Value^*b*^ for:		SEM^*c*^	*P* value
	P_A_	P_B_		
pH	5.68	5.85	0.060	0.179
DM (g/kg FM)	166 B	207 A	9.288	<0.001
WSC (g/kg DM)	85.7 B	142 A	12.65	<0.001
BC (meq/kg DM)	39.6 A	30.8 B	2.249	0.022
NDF (g/kg DM)	535 B	587 A	12.98	0.018
ADF (g/kg DM)	296 B	324 A	10.46	0.043
CP (g/kg DM)	146 A	108 B	8.933	0.002
LAB (log_10_ CFU/g FM)	4.60 B	5.58 A	0.260	0.014
Aerobic bacteria (log_10_ CFU/g FM)	5.85 A	7.93 B	0.439	<0.001
Yeasts (log_10_ CFU/g FM)	4.76 B	5.60 A	0.222	0.013
Molds (log_10_ CFU/g FM)	4.60	5.03	0.160	0.597
Enterobacteria (log_10_ CFU/g FM)	5.70 B	7.08 A	0.321	<0.001

a DM, dry matter; FM, fresh material; WSC, water-soluble carbohydrates; BC, buffering capacity; NDF, neutral detergent fiber; ADF, acid detergent fiber; CP, crude protein; LAB, lactic acid bacteria.

b Means followed by different letters in the same row differ at a *P* of <0.05. P_A_, *P. giganteum* harvested at the early vegetative stage; P_B_, *P. giganteum* harvested at the late vegetative stage.

c SEM, standard error of the mean.

### Fermentation quality of ensiled *P. giganteum*.

Growth stage significantly (*P* < 0.05) affected the pH value, the concentration of lactic acid (LA), and the ratio of LA to acetic acid (AA), while fermentation time significantly (*P < *0.05) affected the pH value, the concentrations of LA, AA, and propionic acid (PA), and the LA/AA ratio (Table 2). The pH of silage from natural fermentation of *P. giganteum* harvested at the early vegetative stage (NP_A_) did not change within the initial 7 days of ensiling and then decreased significantly (*P < *0.05), reaching the lowest value of 4.03 on day 30 of ensiling. In contrast, the pH of silage from the natural fermentation of *P. giganteum* harvested at the late vegetative stage (NP_B_) sharply (*P < *0.05) decreased during the first 7 days of ensiling, with the lowest value (3.50) on day 30 of ensiling, and then increased slightly (*P > *0.05). During the whole ensiling, NP_B_ always had a lower pH value than NP_A_ (*P < *0.05). The LA concentration presented the opposite trend to the pH value, with the highest concentrations of 42.3 and 74.1 g/kg DM on day 30 of ensiling in NP_A_ and NP_B_, respectively. Regardless of growth stage, the AA concentration increased with the extension of fermentation time. Throughout the ensiling process, the LA/AA value showed an upward and then downward tendency, with the maximum on day 30 of ensiling. Butyric acid (BA) was not detected in any *P. giganteum* silages except 60-day NP_A_.

**TABLE 2:** Effects of growth stage and fermentation time on fermentation performance of ensiled *P. giganteum*

Parameter^*a*^	Treatment^*b*^	Value^*c*^ at indicated fermentation time (days)						SEM^*d*^	*P* value^*e*^		
		1	3	7	15	30	60		*G*	*T*	*G* × *T*
pH	NP_A_	5.57	5.64 A	5.62 A	4.76 A	4.03 A	4.16 A	0.139	<0.001	<0.001	<0.001
	NP_B_	5.54	4.66 B	3.87 B	3.84 B	3.50 B	3.55 B				
DM (g/kg FM)	NP_A_	160 B	152 B	148 B	146 B	143 B	131 B	3.654	<0.001	0.020	0.976
	NP_B_	197 A	185 A	180 A	175 A	177 A	171 A				
LA (g/kg DM)	NP_A_	ND	0.37	0.87 B	15.7 B	42.3 B	30.0 B	4.250	<0.001	<0.001	<0.001
	NP_B_	0.21	10.6	31.3 A	51.0 A	74.1 A	59.8 A				
AA (g/kg DM)	NP_A_	2.54 A	5.44	3.64 B	12.6 B	21.2	29.7 A	1.638	0.045	<0.001	<0.001
	NP_B_	0.10 B	4.77	11.8 A	19.3 A	19.6	25.9 B				
LA/AA	NP_A_	–	0.07	0.23 B	1.25	2.01	1.01 B	0.212	<0.001	<0.001	0.025
	NP_B_	2.00	2.02	2.72 A	2.70	3.87	2.32 A				
PA (g/kg DM)	NP_A_	0.35	1.84 A	1.53 A	0.94	1.41	0.17	0.121	0.083	0.007	0.298
	NP_B_	0.23	0.77 B	0.64 B	1.24	1.00	0.24				
BA (g/kg DM)	NP_A_	ND	ND	ND	ND	ND	0.10	–	–	–	–
	NP_B_	ND	ND	ND	ND	ND	ND				
WSC (g/kg DM)	NP_A_	82.1 B	80.5 B	77.1 B	67.4	50.2 B	41.6 B	4.288	<0.001	<0.001	<0.001
	NP_B_	132 A	119 A	91.3 A	76.9	66.3 A	57.0 A				
NH_3_-N (g/kg TN)	NP_A_	73.5 A	82.8 A	92.0 A	119	125	167 A	6.722	<0.001	<0.001	0.825
	NP_B_	37.1 B	51.9 B	62.9 B	88.1	81.6	100 B				

a DM, dry matter; FM, fresh material; LA, lactic acid; AA, acetic acid; LA/AA, ratio of lactic acid to acetic acid; PA, propionic acid; BA, butyric acid; WSC, water-soluble carbohydrates; NH_3_-N, ammonia-nitrogen; TN, total nitrogen; ND, not detected.

b NP_A_, natural fermentation of *P. giganteum* harvested at the early vegetative stage; NP_B_, natural fermentation of *P. giganteum* harvested at the late vegetative stage.

c Means followed by different letters in the same column differ at a *P* of <0.05.

d SEM, standard error of the mean.

e *G*, effect of growth stage; *T*, effect of fermentation time; *G* × *T*, interaction between growth stage and fermentation time.

The DM and WSC content and the ammonia-nitrogen (NH_3_-N) concentration were affected by growth stage and fermentation time, and the WSC content was affected by their interaction (*P < *0.05). As ensiling proceeded, the DM and WSC content decreased and the NH_3_-N concentration increased (*P < *0.05). NP_B_ had higher DM and WSC contents but a lower NH_3_-N concentration than NP_A_.

For microbial counting, growth stage significantly (*P < *0.05) affected the number of LAB, while fermentation time and their interaction significantly (*P < *0.05) affected the number of LAB, aerobic bacteria, and yeasts in *P. giganteum* silage (Table 3). The number of LAB in NP_A_ and NP_B_ showed a downward trend after an initial rise, but the numbers of aerobic bacteria, yeasts, and molds constantly decreased to low or undetected levels. The number of LAB in NP_A_ increased slowly during the first 7 days of ensiling but then increased significantly (*P < *0.05), and the maximum (7.94 log_10_ CFU/g of fresh material [FM]) was detected on day 30 of ensiling. In contrast, the number of LAB in NP_B_ increased rapidly within the initial 3 days of ensiling, peaked on day 15 of ensiling, and then decreased gradually. After 60 days of ensiling, NP_B_ had more LAB but fewer yeasts than NP_A_.

**TABLE 3:** Effects of growth stage and fermentation time on the microbial number of ensiled *P. giganteum*

Parameter^*a*^	Treatment^*b*^	Value^*c*^ at indicated fermentation time (days)						SEM^*d*^	*P* value^*e*^		
		1	3	7	15	30	60		*G*	*T*	*G* × *T*
LAB (log_10_ CFU/g FM)	NP_A_	3.80 B	4.53 B	4.68 B	6.37 B	7.94	7.07 B	0.289	<0.001	<0.001	<0.001
	NP_B_	5.77 A	7.52 A	8.38 A	9.01 A	8.70	8.10 A				
Aerobic bacteria (log_10_ CFU/g FM)	NP_A_	5.48 B	4.34	3.50	2.63	<2.00	2.60	0.356	0.110	<0.001	<0.001
	NP_B_	7.47 A	4.79	3.64	2.70	<2.00	<2.00				
Yeasts (log_10_ CFU/g FM)	NP_A_	4.34	4.27	4.30	4.04	3.47 A	3.52 A	0.146	0.842	<0.001	0.001
	NP_B_	5.40	5.15	4.05	3.88	2.70 B	2.60 B				
Molds (log_10_ CFU/g FM)	NP_A_	4.54 B	4.50 A	3.88	3.04	ND	ND				
	NP_B_	4.96 A	2.94 B	ND	ND	ND	ND				
Enterobacteria (log_10_ CFU/g FM)	NP_A_	5.71 B	5.56 A	5.29	3.81 A	<2.00	2.60				
	NP_B_	6.77 A	4.56 B	2.37	<2.00 B	ND	ND				

a LAB, lactic acid bacteria; FM, fresh material.

b NP_A_, natural fermentation of *P. giganteum* harvested at the early vegetative stage; NP_B_, natural fermentation of *P. giganteum* harvested at the late vegetative stage.

c Means followed by different letters in the same column differ at a *P* of <0.05. ND, not detected.

d SEM, standard error of the mean.

e *G*, effect of growth stage; *T*, effect of fermentation time; *G* × *T*, interaction between growth stage and fermentation time.

### The bacterial community of fresh and ensiled *P. giganteum*.

**FIG 1:**
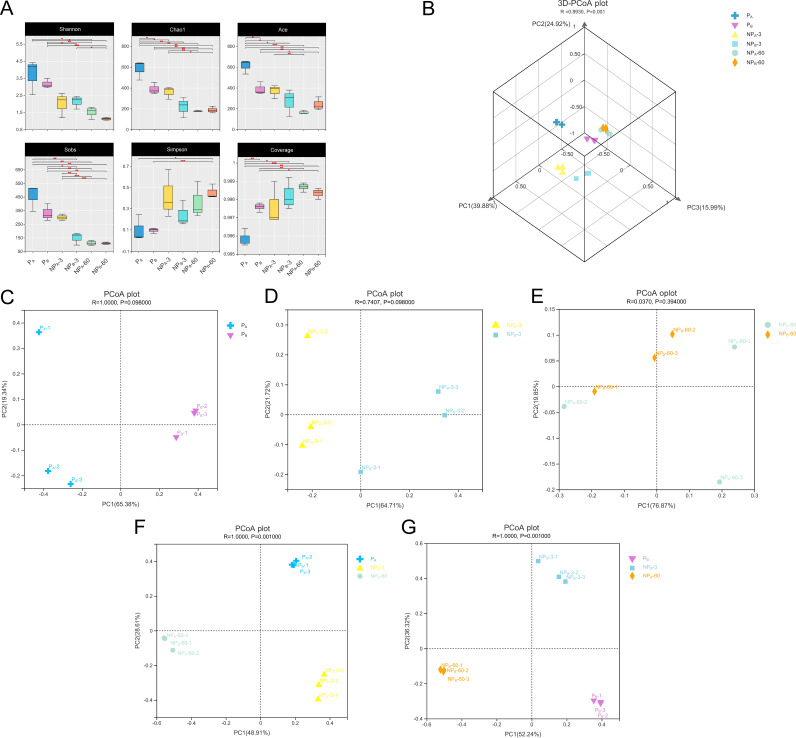
Bacterial community diversities of fresh and ensiled *P. giganteum* harvested at different growth stages. (A) Alpha diversities (Shannon, Chao1, ACE, Sobs, Simpson, and coverage indexes) of bacterial community. (B) Beta diversities of bacterial community, calculated by three-dimensional principal-coordinate analysis (3D-PCoA) based on the Bray-Curtis distance metric. (C to E) PCoA analyses after 0, 3, and 60 days of ensiling, respectively. (F and G) PCoA analyses during ensiling of P_A_ and P_B_, respectively.

The alpha diversities of the bacterial community in fresh and ensiled *P. giganteum* are presented in Fig. 1A. The indexes of Shannon, Chao1, abundance-based coverage estimator (ACE), and Sobs were highest in fresh sample of *P. giganteum* at the early vegetative stage (P_A_), followed by fresh sample of *P. giganteum* at the late vegetative stage (P_B_) and then ensiled samples. Among all silages, 60-day *P. giganteum* silages (NP_A_-60 and NP_B_-60) had the lowest Shannon, Chao1, ACE, and Sobs indexes. The average coverage index of all samples was above 0.995. The beta diversities of the bacterial communities in fresh and ensiled *P. giganteum* presented by the Bray-Curtis distance metric are shown in Fig. 1B. A clear separation was observed between the symbols of fresh and ensiled samples in the three-dimensional principal-coordinate analysis (3D-PCoA) plot. Among them, those for P_A_ and P_B_ as well as NP_A_-3 and NP_B_-3 were well separated in Fig. 1C and D, while those for P_A_, NP_A_-3, and NP_A_-60 as well as P_B_, NP_B_-3, and NP_B_-60 were well separated in Fig. 1F and G. Overall, the symbols for 60-day ensiled samples (NP_A_-60 and NP_B_-60) clustered together (Fig. 1B and E).

**FIG 2:**
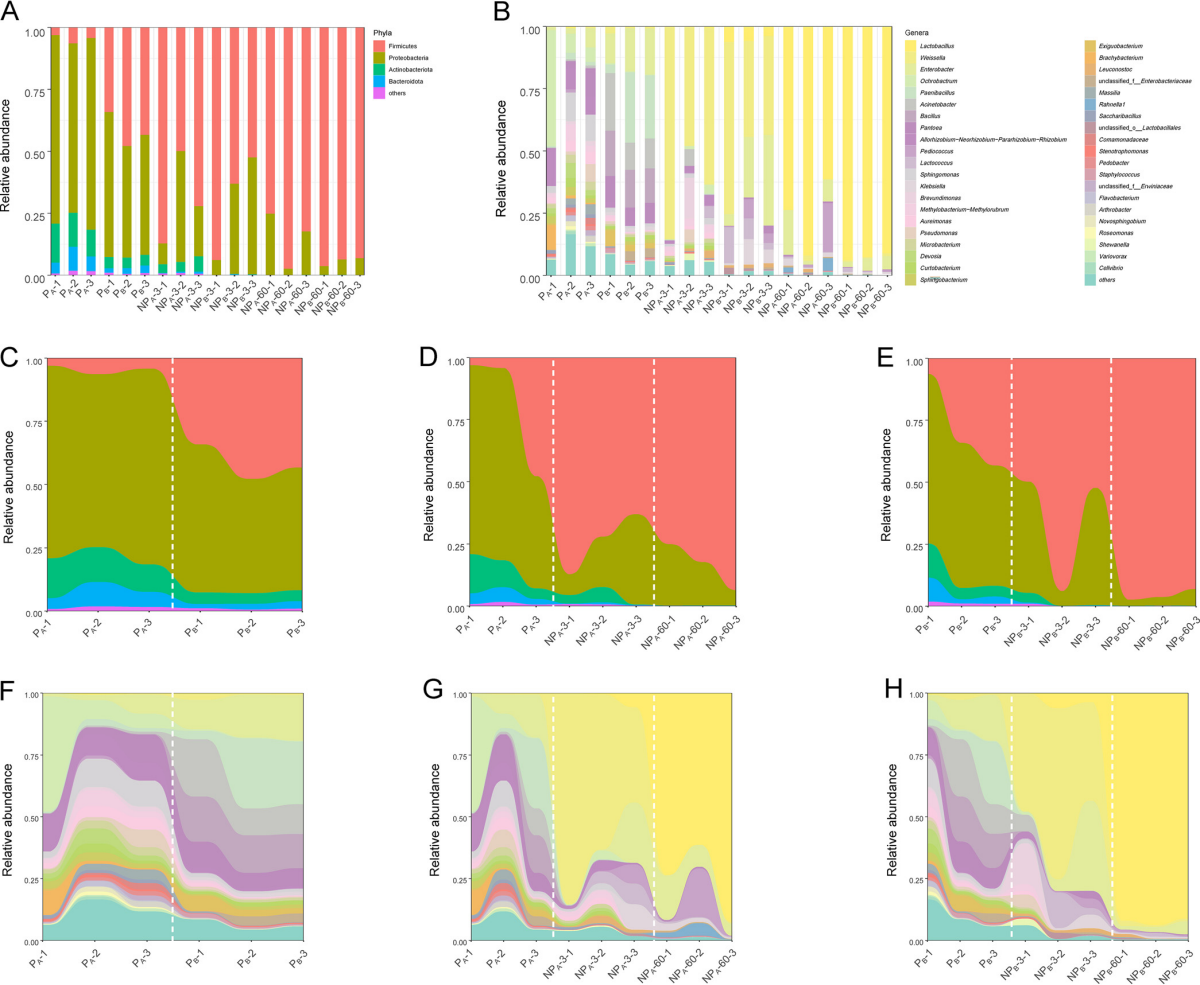
Bacterial community composition and succession of fresh and ensiled *P. giganteum* harvested at different growth stages. (A) Relative abundance of bacterial community at the phylum level. (B) Relative abundance of bacterial community at the genus level. (C and F) Bacterial community successions during the growth of fresh *P. giganteum* are aggregated and colored on a stream graph by phylum and genus, respectively. (D and G) Bacterial community successions during ensiling of P_A_ are aggregated and colored on a stream graph by phylum and genus, respectively. (E and H) Bacterial community successions during ensiling of P_B_ are aggregated and colored on a stream graph by phylum and genus, respectively. The numbered treatments (e.g., P_A_-1, P_A_-2, and P_A_-3) are replicates.

As shown in Fig. 2A, *Proteobacteria* (73.9%) was the abundant phylum in P_A_, yet *Proteobacteria* (50.7%) and *Firmicutes* (41.8%) were the phyla with high relative abundances in P_B_. As *P. giganteum* grew, the relative abundances of *Proteobacteria*, *Actinobacteriota*, and *Bacteroidota* decreased from 73.9%, 13.4%, and 6.66% to 50.7%, 4.38%, and 2.32%, respectively, but *Firmicutes* increased from 4.65% to 41.8%. After 3 days of ensiling, the relative abundance of *Firmicutes* in NP_A_ and NP_B_ increased in various degrees, accompanied by a decrease of the relative abundances of *Proteobacteria*, *Actinobacteriota*, and *Bacteroidota*. After 60 days of ensiling, *Firmicutes* were predominant (>85%) in both NP_A_ and NP_B_ silages.

The numbers of genera with a relative abundance greater than 1% in P_A_ and P_B_ were 18 and 12, respectively (Fig. 2B). The most abundant genus in P_A_ was *Ochrobactrum* (20.0%), followed by *Rhizobium* (10.1%) and *Sphingomonas* (8.18%), while *Paenibacillus* (19.0%), *Bacillus* (15.9%), Enterobacter (15.9%), Acinetobacter (15.3%), and *Pantoea* (7.92%) were the 5 genera with high relative abundances in P_B_. From P_A_ to P_B_, the relative abundances of Enterobacter, *Bacillus*, and Acinetobacter increased from 3.74%, <1.00%, and <1.00% to 15.9%, 15.7%, and 15.3%, but *Rhizobium*, *Aureimonas*, and *Sphingobacterium* decreased from 10.1%, 3.84%, and 2.65% to 1.44%, 0.65%, and 1.11%, respectively. Moreover, the relative abundances of *Aureimonas*, *Microbacterium*, and *Saccharibacillus* decreased to below 1.00%. With the process of ensiling, the relative abundance of *Ochrobactrum*, Acinetobacter, and *Sphingomonas* decreased to an undetectable level, while after 60 days of ensiling, *Lactobacillus* dominated the bacterial community of both NP_A_ and NP_B_, with relative abundance accounting for 75.7% and 92.6%, respectively.

The stream graph showed that both growth stage and storage time had a remarkable impact on the succession of bacterial communities in *P. giganteum* silage (Fig. 2C to H). The variation in growth stage from the early vegetative stage to the late vegetative stage affected the bacterial community succession of *P. giganteum* (Fig. 2C and F), while the ensiling process had a more significant effect on the bacterial community succession (Fig. 2D, E, G, and H).

### Bacterial cooccurrence networks of fresh and ensiled *P. giganteum*.

**FIG 3:**
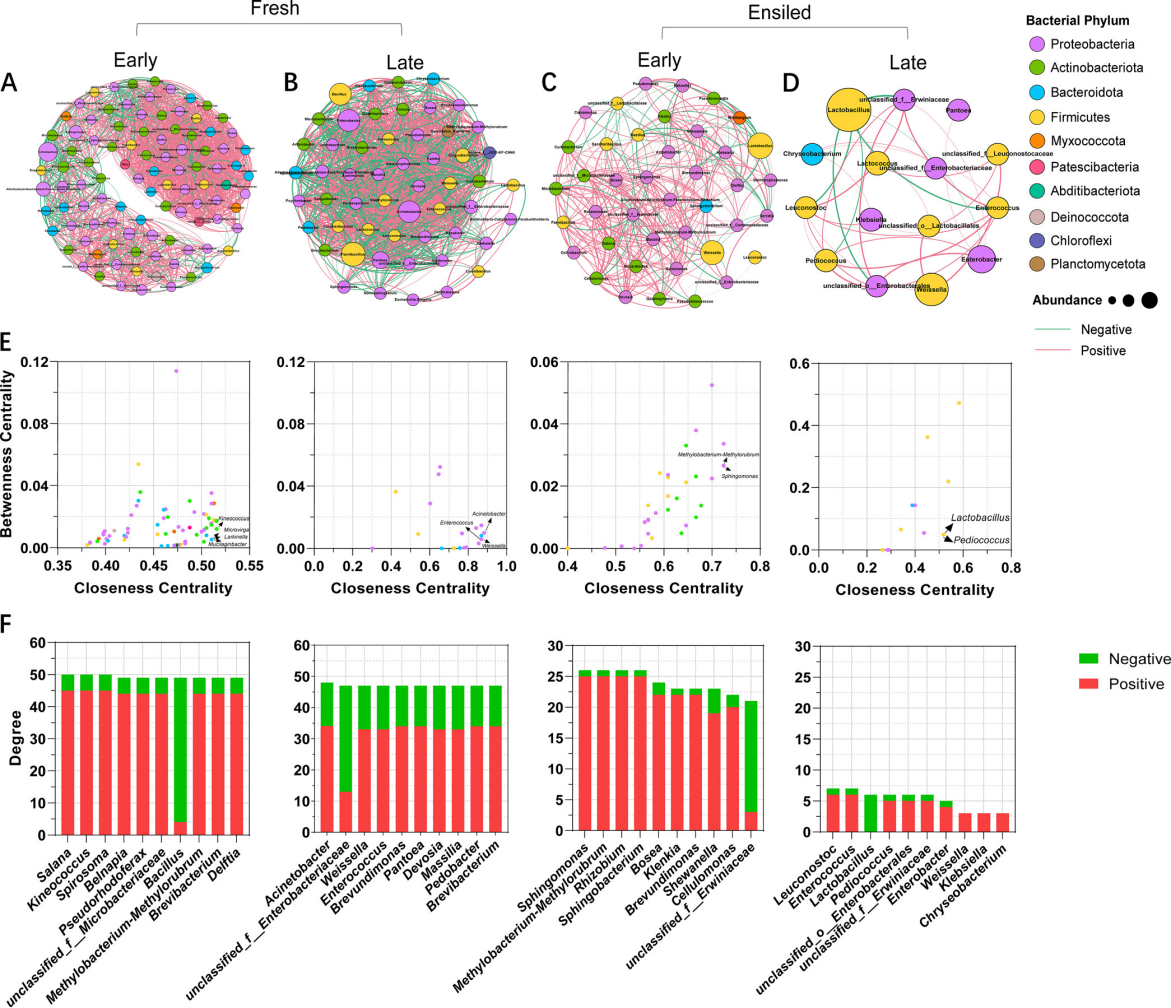
Bacterial cooccurrence network analyses of fresh and ensiled *P. giganteum* harvested at different growth stages. Bacterial cooccurrence networks of P_A_ (A), P_B_ (B), NP_A_ (C), and NP_B_ (D). Node colors indicate bacterial phyla, and node sizes indicate their relative abundance. Edges are colored according to negative (green) and positive (red) correlations. (E) Comparison of node-level topological features (betweenness centrality and closeness centrality) in four networks. (F) Interaction type of the top 10 hub nodes with high degree.

The cooccurrence network based on the correlation coefficient matrix can reflect, to a certain extent, the relationships between microbial members. Thus, the bacterial cooccurrence networks of fresh and ensiled *P. giganteum* based on Spearman’s rank correlation were separately created at two growth stages to clearly understand the effects of growth stage on the interrelationships of bacterial members (genera). Based on cooccurrence network analysis (Fig. 3A to D and Table 4), the numbers of nodes and edges in bacterial networks were ranked as follows: P_A_ > P_B_ > NP_A_ > NP_B_. The taxa with low betweenness centrality and high closeness centrality were *Kineococcus*, *Larkinella*, *Microvirga*, and *Mucilaginbacter* in P_A_, Acinetobacter, *Weissella*, *Enterococcus*, and *Stenotrophomonas* in P_B_, *Sphingomonas* and *Methylobacterium-Methylorubrum* in NP_A_, and *Lactobacillus* and *Pedicoccus* in NP_B_ (Fig. 3E). Further, the edges of the top 10 nodes with high degrees were primarily positive with other nodes in the bacterial networks of fresh and ensiled samples (Fig. 3F).

**TABLE 4:** Network topological characteristics of fresh and ensiled *P. giganteum*^*a*^

Parameter	Value for:			
	P_A_	P_B_	NP_A_	NP_B_
No. of nodes	100	54	41	15
No. of edges	1,747	1,083	341	31
Avg degree	34.9	40.1	16.6	4.13
Clustering coefficient	0.803	0.924	0.677	0.679

a P_A_, *P. giganteum* harvested at the early vegetative stage; P_B_, *P. giganteum* harvested at the late vegetative stage; NP_A_, natural fermentation of *P. giganteum* harvested at the early vegetative stage; NP_B_, natural fermentation of *P. giganteum* harvested at the late vegetative stage.

### Correlation analysis of chemical composition and phyllosphere microbiota as well as fermentation parameters and silage microbiota.

**FIG 4:**
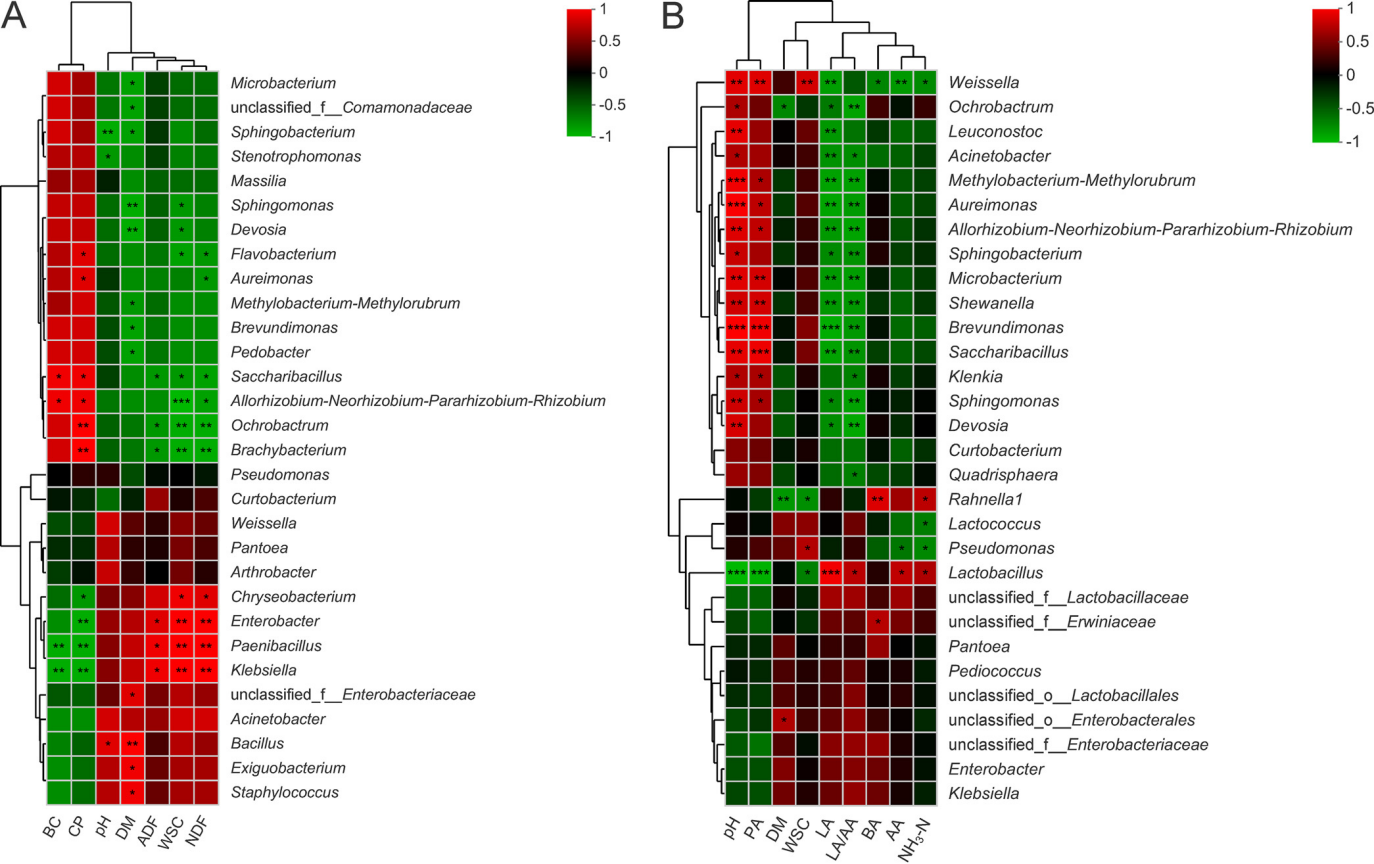
Spearman’s correlation heatmap of chemical composition and phyllosphere microbiota (top 30 genera) in fresh *P. giganteum* (A) and fermentation parameters and silage microbiota (top 30 genera) in ensiled *P. giganteum* (B). Red squares indicate positive correlations (0 < *R* < 1), whereas green squares indicate negative correlations (−1 < *R* < 0). *, *P < *0.05; **, 0.001 < *P ≤ *0.01.

In fresh *P. giganteum* (Fig. 4A), *Microbacterium*, *Sphingobacterium*, *Sphingomonas*, and *Methylobacterium-Methylorubrum*, etc., were negatively (*P < *0.05) related to DM, with correlation coefficients of −0.891, −0.886, −0.943, and −0.829, respectively. *Rhizobium*, *Ochrobactrum*, and *Brachybacterium* were positively (*P < *0.05) correlated with CP (*R* = 0.881, 0.933, and 0.938, respectively) but negatively (*P < *0.05) correlated with WSC (*R* = −1.00, −0.939, and −0.940, respectively). Conversely, *Paenibacillus* and Enterobacter were negatively (*P < *0.01) correlated with CP (*R* = −0.936 and −0.942) but positively (*P < *0.01) correlated with WSC (*R* = 0.930 and 0.943) and NDF (*R* = 0.936 and 0.931). *Bacillus* was positively correlated with pH (*R* = 0.829, *P < *0.05) and DM (*R* = 0.943, *P < *0.01). In ensiled *P. giganteum* (Fig. 4B), Enterobacter and *Pantoea* were positively (*P < *0.05) correlated with DM (*R* = 0.503 and 0.618) and WSC (*R* = 0.632 and 0.643). There were negative correlations between pH and *Pantoea* (*R* = −0.610, *P < *0.01) and positive correlations between pH and *Exiguobacterium* (*R* = 0.495, *P < *0.05). *Lactobacillus* was positively correlated with LA (*R* = 0.846, *P < *0.001), AA (*R* = 0.671, *P < *0.05), and the LA/AA ratio (*R* = 0.650, *P < *0.05) but negatively correlated with pH (*R* = −0.895, *P < *0.001), PA (*R* = −0.865, *P < *0.001), and WSC (*R* = −0.622, *P < *0.05). A slight negative correlation (*R* = −0.084, *P > *0.05) was observed between Enterobacter and NH_3_-N concentration.

### KEGG functional profiles of bacterial community.

**FIG 5:**
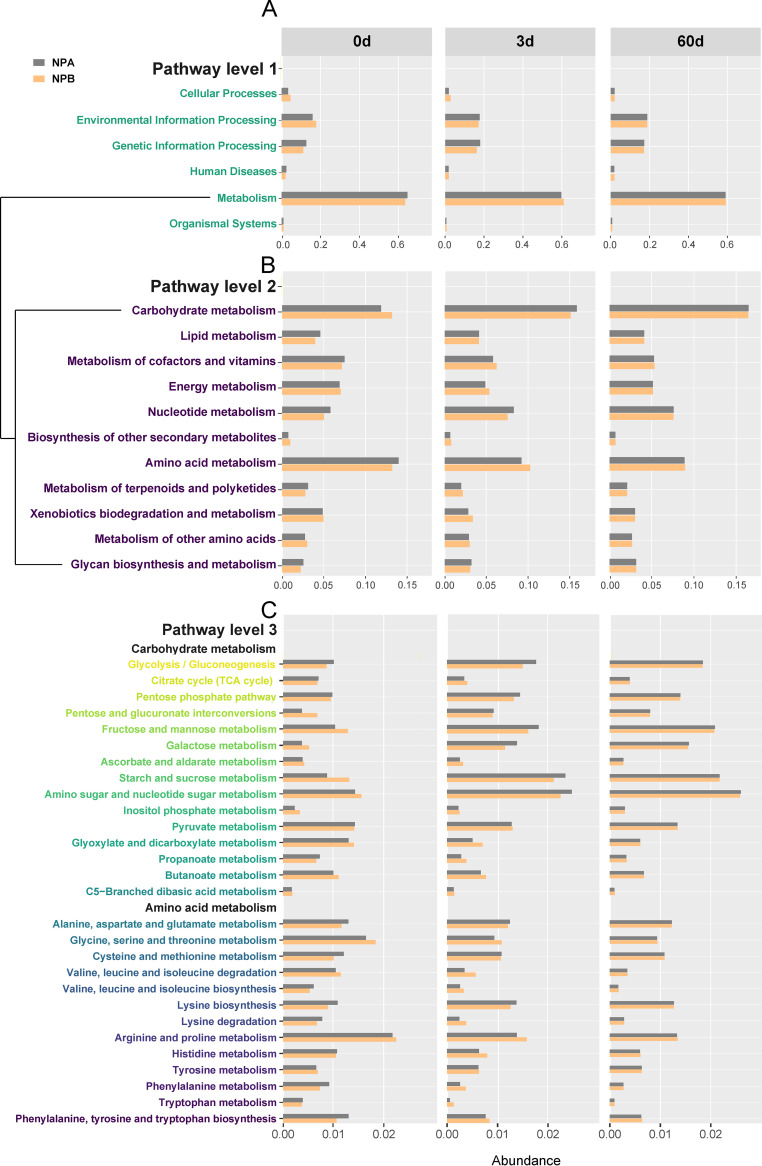
Bacterial functional profiles of fresh and ensiled *P. giganteum* harvested at different growth stages. (A) 16S rRNA gene-predicted KEGG function profiles at pathway level 1. (B) 16S rRNA gene-predicted KEGG function profiles at pathway level 2. (C) 16S rRNA gene-predicted KEGG function profiles at pathway level 3.

As shown in Fig. 5, the KEGG function profiles under pathway level 1 in fresh and ensiled *P. giganteum* were associated primarily with metabolism (Fig. 5A), and the function profiles under pathway level 2 were related mainly to carbohydrate and amino acid metabolism, followed by nucleotide cofactors, vitamins, energy, and lipid metabolism (Fig. 5B). Specifically, P_B_ had a higher abundance of carbohydrate and energy metabolism but a lower abundance of nucleotide, cofactors, vitamins, and lipid metabolism than P_A_. However, there were no differences in those functional categories between NP_A_-60 and NP_B_-60. NP_A_-60 and NP_B_-60 had the highest abundance of carbohydrate metabolism, followed by NP_A_-3 and NP_B_-3 and then P_A_ and P_B_, whereas the abundance of amino acid metabolism exhibited the opposite tendency, with the highest value in P_A_ and P_B_ and the lowest value in NP_A_-60 and NP_B_-60.

Under pathway level 3 (Fig. 5C), the abundance of carbohydrate metabolisms, including pentose and glucuronate interconversions and fructose, mannose, galactose, ascorbate, aldarate, starch, sucrose, amino sugar, nucleotide sugar, inositol phosphate, glyoxylate, dicarboxylate, and butanoate metabolism, was increased in P_B_ compared with P_A_, while the abundance of glycolysis/gluconeogenesis, citrate cycle (tricarboxylic acid [TCA] cycle), pentose phosphate pathway (PPP), and propanoate was decreased in P_B_ in comparison with P_A_. The abundance of amino acid metabolisms, including valine, leucine, and isoleucine degradation and glycine, serine, threonine, arginine, proline, and tyrosine metabolism, was increased in P_B_ compared with P_A_, while the abundance of valine, leucine, isoleucine, lysine, phenylalanine, tyrosine, and tryptophan biosynthesis, lysine degradation, and alanine, aspartate, glutamate, cysteine, methionine, histidine, phenylalanine, and tryptophan metabolism was decreased in P_B_ in comparison with P_A_. For carbohydrate metabolism, NP_A_ and NP_B_ had a higher abundance of glycolysis/gluconeogenesis, PPP, pentose and glucuronate interconversions, fructose, mannose, galactose, starch, sucrose, amino sugar, and nucleotide sugar metabolism but a lower abundance of TCA cycle, ascorbate, aldarate, pyruvate, glyoxylate, dicarboxylate, propanoate, butanoate, butanoate, and C5-branched dibasic acid metabolism than P_A_ and P_B_. For amino acid metabolism, NP_A_ and NP_B_ had a higher abundance of lysine biosynthesis but a lower abundance of glycine, serine, threonine, arginine, proline, histidine, tyrosine, phenylalanine, and tryptophan metabolism, lysine degradation, and valine, leucine, and isoleucine biosynthesis and degradation than P_A_ and P_B_.

## DISCUSSION

The phyllosphere, the aerial parts of plants, is composed mainly of leaves and stems. The phyllosphere microbiota and chemical parameters change greatly as the plants develop and age. Forage maturity is the most important factor affecting their quality at harvest (13). In this work, with the growth of *P. giganteum*, the CP content decreased, while the DM, WSC, NDF, and ADF content increased, which was similar to the findings of Xue et al. (14). The increase in DM content could be attributed to the accumulation of carbohydrates (nonstructural or structural carbohydrates). The DM content of *P. giganteum* was low (<25%) even when harvested at the late vegetative stage, which was related to the physiological characteristics of tropical grass. Oliveira et al. (6) concluded that tropical grass has several unfavorable aspects for silage fermentation, including a low DM content at harvest. The increase in NDF and ADF content could be explained by the increase in the stem-to-leaf ratio and the accumulation of cell wall (14), since the cell wall components in the stem are higher than those in the leaf (15). Meanwhile, the increased proportion of NDF and ADF led to a relative decrease in CP content (16). The CP content of forage is known to affect its BC, and the decrease in CP content might explain the decrease in BC.

The number of LAB, aerobic bacteria, yeasts, and enterobacteria increased as *P. giganteum* matured, which was in line with the findings of Comino et al. (17). As reported by Macarisin et al. (18), the bacterial number and diversity can be sharply influenced by the growth stages of leaf and plant. Throughout the growth cycle of forage, the external environment (e.g., solar radiation, temperature, and rainfall) and internal environment (e.g., plant morphology, moisture content, and leaf thickness) are constantly changing, and they have been reported to affect microbial colonization (19). Recent studies showed that sugar and volatile organic compounds secreted by forage play an important role in determining the microbial population of forage (20, 21). It is known that microbes, including LAB, are enriched in sugar-rich plants. Thus, the higher WSC content in P_B_ could explain the increased number of phyllosphere microorganisms. Moreover, the nutrient release from aging tissue and leaves in P_B_ was also considered to be beneficial to the growth of microorganisms (22).

Kung and Shaver (23) indicated that the pH value of high-quality silage should be below 4.20 within 21 days of ensiling. Therefore, the rate of decline of pH during ensiling has been used to determine whether the silage is effectively preserved. The slower the decline of the pH value, the greater the risk of undesired microbial activities and nutritional losses in silage. In this work, the pH of NP_A_ during the initial phase of ensiling was difficult to decrease due to the low LAB number (<5.0 log_10_ CFU/g of FM) of P_A_, while the lower pH and higher LA concentration in NP_B_ than in NP_A_ could be related to the higher WSC content, sufficient LAB number, and lower BC of P_B_. Interestingly, on day 1 of ensiling, AA but no LA was detected in NP_A_. This finding is consistent with the finding of Kung and Shaver (23) that AA is the first organic acid produced during ensiling. The above-mentioned study suggested that the generation of AA in the initial phase of ensiling could reduce the silage pH to about 5.0, which was helpful to establish a weak acid environment. As in most studies, AA concentration increased with the extension of ensiling, and this could be associated with the activities of AA-producing microbes and the shift from homotypic fermentation to heterotypic fermentation. Clostridia are generally found in silage with less than 30% DM (8). The production of BA is closely associated with clostridial growth. But no or negligible BA was detected in NP_A_ and NP_B_, indicating that clostridial fermentation did not occur in *P. giganteum* silage.

The NH_3_-N concentration has long been used to evaluate the protein degradation degree and fermentation quality of silage. The concentration of NH_3_-N is commonly expressed as a percentage of total nitrogen (TN) (or per kilogram of TN), and it should be less than 10% (or 100 g/kg) of TN in high-quality silage (5). The final NH_3_-N concentration of NP_A_ (167 g/kg TN) was obviously higher than the above-mentioned limit, suggesting that serious proteolysis occurred in *P. giganteum* silage harvested at the early vegetative stage. The protein degradation and ammonia generation in silage comprise a complex biochemical process, involving the activities of plant protease, clostridia, and enterobacteria, etc. (5). The degradation degree of silage protein depends on the decline rate of pH during ensiling (8). The slow pH decline of NP_A_ in the initial phase of ensiling could not inhibit the degradation and deamination of protein effectively, resulting in the high NH_3_-N concentration of NP_A_. Furthermore, the high moisture content in P_A_ might also contribute to the high NH_3_-N concentration in NP_A_, as studies have shown that high moisture could benefit the activity of plant proteases (24). Unlike with NP_A_, the NH_3_-N concentration of NP_B_ was maintained at a low level (<100 g/kg TN) throughout ensiling, which could be ascribed to the rapid reduction of pH in the first 7 days of ensiling.

The higher number of LAB in NP_B_ was probably because the high WSC content of P_B_ (142 g/kg DM) accelerated the growth of phyllosphere LAB. As ensiling proceeded, the number of aerobic bacteria and molds in both treatments rapidly decreased to an undetectable level, and the reason could be speculated to be the depletion of oxygen. And the negligible number of yeasts and enterobacteria in NP_B_ was associated with its low pH value. Further, the decrease of yeasts in NP_B_ was also related to the rapid proliferation of LAB, as the former are less competitive under anaerobic and acidic conditions (5).

The average coverage index in all sequenced samples was above 99%, indicating that the sequencing depth was sufficient for reliable analysis of the bacterial community. The higher number of operational taxonomic units (OTUs) in P_A_ than in P_B_ reflected a richer bacterial composition in *P. giganteum* harvested at the early vegetative stage. Bacterial alpha diversity, characterized by Chao1 and Shannon indexes, decreased with the maturity of *P. giganteum*. This could be explained by several factors, such as complex climate conditions (UV radiation exposure, precipitation, and temperature, etc.) and low water and nitrogen availability on the plant surface. Ensiling further decreased the bacterial alpha diversity of *P. giganteum*, which could be associated with the disappearance of acid-intolerant and anaerobically intolerant phyllosphere bacteria (25). Low pH conditions are mainly responsible for the reduced microbial diversity in acidic habitats (26). The high WSC content and sufficient LAB number of P_B_ ensured adequate LAB proliferation and rapid acidification during ensiling, thereby reducing the bacterial alpha diversities of NP_B_, which in turn explained the lowest indexes of Chao1 and Shannon in NP_B_ with the lowest pH value.

The PCoA plot was made to visualize bacterial community composition differences as distances between treatments. The clear separation of symbols between P_A_ and P_B_ indicated great differences in bacterial community composition of fresh *P. giganteum* harvested at two growth stages, and this discrepancy could be associated with the chemical parameters of forage, climate, or other factors. Meanwhile, the separated clustering between the fresh and ensiled *P. giganteum*, as mentioned above, was attributed to the disappearance of aerobically and acid-intolerant phyllosphere bacteria during ensiling (25). The decrease of the distance between the symbols from day 3 to day 60 showed that as ensiling proceeded, the bacterial community compositions of ensiled *P. giganteum* tended to be similar. Although great differences were found in the phyllosphere microbiota and chemical parameters of fresh *P. giganteum* at two growth stages, the ensiling process seems to weaken the effect of growth stage on bacterial community composition of the silage. The distance between P_A_, NP_A_-3, and NP_A_-60 or P_B_, NP_B_-3, and NP_B_-60 was larger than that between P_A_ and P_B_, suggesting that although growth stage and fermentation time both affected the bacterial community effectively, fermentation time seemed to have a greater influence than growth stage on the bacterial community compositions of *P. giganteum* silage.

This obvious shift of bacterial community from *Proteobacteria* to *Firmicutes* before and after ensiling could be ascribed to the inhibition of aerobic bacteria (Acinetobacter, *Sphingomonas*, and Pseudomonas, etc.) and the thriving of LAB (*Weissella*, *Pediococcus*, and *Lactobacillus*). The ensiling environment favors the growth of *Firmicutes* because they are typical microbes under anaerobic and acid conditions (27). Similar to the findings of Pang et al. (28), *Lactobacillus*, *Weissella*, and *Pediococcus* were the three most dominant genera during ensiling in this work. *Weissella* and *Pediococcus* are generally considered early colonizers during ensiling (29, 30) due to their weaker tolerance to acid than that of *Lactobacillus*. But the initial acid environment established by *Pediococcus* and *Weissella* is suitable for the growth of *Lactobacillus* (31). Thus, with the process of ensiling, the bacterial community of NP_A_ and NP_B_ was first dominated by *Weissella* (day 3) and finally dominated by *Lactobacillus* (day 60).

Cooccurrence networks among bacterial genera for fresh and ensiled *P. giganteum* were constructed to clearly understand the effects of growth stage and fermentation time on the correlation and interaction of the phyllosphere and the silage microbiome. Based on the number of nodes and edges, the complexity of bacterial networks in fresh *P. giganteum* decreased from the early vegetative stage to the late vegetative stage (Fig. 2A and B). Ensiling further decreased the complexity of bacterial networks, with the simplest bacterial correlation structures in NP_B_ (Fig. 2C and D). High fermentation quality was accompanied by low bacterial network complexity, which is in line with the findings of Bai et al. (32).

As reported by Banerjee et al. (33), a negative correlation of cooccurrence networks indicates a possible competition for resources and common predators, while a positive correlation indicates symbiotic or cooperative relationships within microbial taxa. The higher proportion of negative correlations in the network of P_B_ (39.8%) than in the network of P_A_ (21.6%) showed that with the growth of *P. giganteum*, the competition among bacterial taxa in its phyllosphere microbiota intensified. Furthermore, the higher proportion of positive correlations in the network of NP_A_ and NP_B_ (80.9 and 80.6%) than in the networks of P_A_ and P_B_ (78.4 and 60.2%) reflected the increased cooperative interactions among bacterial taxa after ensiling. Thus, we speculated that the microbiome in silages is primarily cooperative, which suggests that the survival of phyllosphere bacteria after ensiling is related to the cooperation among bacterial cells, and might also be confirmed by findings such as the establishment by *Lactococcus* and *Weissella* of an acidic environment suitable for the growth of *Lactobacillus* (31).

According to Berry and Widder (34), the keystone taxa in the bacterial community can be identified by the combined scores of low betweenness centrality, high closeness centrality, and high mean degree. Correspondingly, *Kineococcus*, *Larkinella*, *Microvirga*, and *Mucilaginbacter* in P_A_, Acinetobacter, *Weissella*, and *Enterococcus* in P_B_, *Sphingomonas* and *Methylobacterium-Methylorubrum* in NP_A_, and *Lactobacillus* and *Pediococcus* in NP_B_ were identified as the keystone taxa (Fig. 2E and F). It should be noted that the keystone taxa in this work were not necessarily the ones with the highest relative abundance. Similarly, previous studies found that although keystone taxa have considerable effects on bacterial community and functions, their abundance is not proportional to their effects (32, 35, 36).

In fresh *P. giganteum*, the significant positive correlation between *Rhizobium* or *Ochrobactrum* and CP content could be explained by their nitrogen-fixing capacity, as these two genera belong to *Rhizobiaceae* (37). In *P. giganteum* silage, *Lactobacillus* was positively correlated with AA concentration, indicating that the production of AA during the ensiling of *P. giganteum* is related to the heterofermentative strains of *Lactobacillus*. The significant negative correlation between *Lactobacillus* and WSC content showed that the decrease of WSC in *P. giganteum* silage is due mainly to the consumption of *Lactobacillus* rather than undesirable microorganisms. In general, plant protease, enterobacteria, and/or clostridia are involved the generation of NH_3_-N during ensiling (5). However, no clostridia were detected in all *P. giganteum* silages. Meanwhile, there was a slight negative correlation between Enterobacter and NH_3_-N concentration. These results suggested that the presence of enterobacteria and clostridia did not provide an explanation of the generation of NH_3_-N in *P. giganteum* silage. The generation of NH_3_-N during the ensiling of *P. giganteum* might be associated primarily with plant protease, but further study is required.

High-throughput sequencing is a powerful tool for assessing microbial community structure and diversity but does not provide insights into the metabolic potential of the community, even though functional diversity is crucial for the analysis of microbial communities (11). Here, the reliable Tax4Fun tool was applied to predict the KEGG functional profiles of the bacterial community in fresh and ensiled *P. giganteum*. Based on the abundance, the functional profiles under pathway level 2 of the microbiota involved in fresh material and silage fermentation are dominated by the metabolism of carbohydrate, amino acid, cofactors, vitamins, energy, and nucleotides, which is in line with the findings of Bai et al. (32). Thompson et al. (22) reported that the leakage of sugars from aging tissues and leaves could promote microbial growth, which might explain the promotion of carbohydrate metabolism of the bacterial community in fresh *P. giganteum* from the early vegetative stage to the late vegetative stage. Also, the extensive utilization of carbohydrates by phyllosphere bacteria to produce extracellular polysaccharides may explain the promotion of carbohydrate metabolism during the late growth stage, since extracellular polysaccharides are reported to play an important role in bacterial adaptation to different stress conditions (such as desiccation) resulting from plant maturation (38), while the suppression of lipid and amino acid metabolism might be attributed to the decrease of lipid and protein during plant maturation (9). In the process of plant senescence, there is not only synthesis of new lipids and proteins but also reduction of lipids and proteins. However, on the whole, the content of lipids and proteins shows a downward trend (39).

The principle of silage fermentation is that the available carbohydrates in forage are fermented to short-chain fatty acids (mainly lactate) by LAB under anaerobic conditions, which may explain the higher abundance of carbohydrate metabolism after ensiling (32). As essential substances in organisms, amino acids play an important role in promoting primary metabolism and protein synthesis. This explains why the abundance of amino acid metabolism in P_A_ is higher than that in P_B_, because P_A_ possesses a richer bacterial composition, while the suppressed amino acid metabolism after ensiling is attributed to its low pH value. A previous study indicated that the acidic environment established by ensiling can inhibit the amino acid metabolism induced by undesirable microorganisms (40). The metabolism of cofactors and vitamins was suppressed during ensiling, which was consistent with the results of Wang et al. (41). This is to be expected, as Liu et al. (42) reported that although vitamins are beneficial to the immune system of ruminants, they can be degraded during ensiling. The ensiling process did not promote energy metabolism, which might be related to the restricted energy metabolism of the undesirable microorganisms that do not tolerate acid and aerobic conditions. Intriguingly, the shifts of nucleotide metabolism during ensiling were opposite to that of energy metabolism. Nucleotides can be used to synthesize DNA and provide major energy for cellular processes (43). Thus, other omics methods, such as proteomics, metagenomics, and metabolomics, are required to further assess the functional annotation and metabolic pathway of the microbial community before and after ensiling.

To further clarify the functional shifts of the bacterial community in fresh and ensiled *P. giganteum*, carbohydrate metabolism and amino acid metabolism were specifically analyzed at pathway level 3. With *P. giganteum* growth, most of the carbohydrate metabolic categories (10 of a total of 15) showed an increasing trend, while most of the amino acid metabolic categories (9 of a total of 13) showed a decreasing trend, which was consistent with the increase in WSC and the decrease in CP during plant growth, indicating that the succession of phyllosphere bacteria was closely related to the change of phyllosphere nutrients. Regardless of growth stage, the difference in carbohydrate and amino acid metabolic categories in ensiled *P. giganteum* was found to be greatest on days 0 and 3, not on day 60. This indicated that carbohydrate and amino acid metabolic activities of the bacterial community varied mostly before and at the beginning of ensiling but stabilized after 60 days of ensiling. Indeed, there should be minimal changes in metabolic activity in silage when silage is well fermented and fermentation enters a stable phase.

The metabolism of fructose, mannose, galactose, sucrose, amino sugar, and nucleotide sugar was promoted with the process of ensiling, which was in accordance with the fact that LAB can utilize a variety of carbohydrate sources for anaerobic fermentation. Among them, these promoted sugar metabolisms in ensiled samples were related primarily to the full proliferation of LAB, represented by *Weissella* and *Lactobacillus*. Differently, the TCA cycle was suppressed by ensiling. The suppression of the TCA cycle after ensiling could be ascribed to the depletion of O_2_ in silos, as this cycle must be performed under aerobic conditions (44). It is known that the Embden-Meyerhof-Parnas (EMP) pathway, the most common type of glycolysis, assists homofermentative LAB to ferment glucose into LA. Hence, we speculated that the higher relative abundance of glycolysis in the late phase of ensiling (day 60) may be related to the abundant homofermentative LAB. The high relative abundances of *Weissella* (day 3) and heterofermentative *Lactobacillus* (day 60) might explain the high abundance of the PPP, because heterofermentative LAB, including *Weissella*, possess the PPP (45). Amino acid metabolism of the bacterial community during ensiling is of significance in explaining the formation of NH_3_-N. And the formation of NH_3_-N in ensiled *P. giganteum* could be due to the promoted metabolism of alanine, aspartate, glutamate, cysteine, and methionine. Threonine, methionine, lysine, histidine, phenylalanine, tryptophan, and phenylalanine are essential amino acids, which animals cannot synthesize (46). The suppressed metabolism and promoted biosynthesis of these essential amino acids in this study suggested an improvement in the nutritional value of *P. giganteum* after ensiling.

Given that these profiles are estimated based on what is commonly observed within a taxonomic group, and that 16S rRNA metabarcoding has very limited taxonomic resolution, these functional profiles are highly speculative and should be examined with caution. The predicted functional profiles should be further validated by transcriptomics, proteomics, and metabolomics studies to provide clear indications.

### Conclusions.

*P. giganteum* contains low BC but high WSC and CP content and can be used as a promising feedstock for anaerobic fermentation. Both growth stage and fermentation time had remarkable effects on fermentation performance, bacterial community, cooccurrence networks, and keystone taxa of *P. giganteum* silage, but fermentation time had a greater influence than growth stage on the bacterial community diversity and functional profiles of *P. giganteum* silage. The 60-day *P. giganteum* silages made at the early and late vegetative stages both displayed the lactate type of fermentation, but the former had a high NH_3_-N concentration. Therefore, *P. giganteum* harvested at the late vegetative stages is recommended for anaerobic fermentation to optimize fermentation quality and yield.

## MATERIALS AND METHODS

### Preparation of materials.

*P. giganteum* was planted in the Baima experimental field of Nanjing Agricultural University (latitude 31.61°N, longitude 119.18°E, elevation of 25.1 m; Jiangsu, China) on 20 May 2020. The experimental field (30 m^2^) was separated equally into three blocks (for replicates), and each block was further divided into two equal plots (for two growth stages). Half of the *P. giganteum* in the plot was mowed on 16 August 2020, and the other half was mowed on 26 September 2020, to obtain two batches of *P. giganteum* (P_A_, early vegetative stage; P_B_, late vegetative stage). The temperature, precipitation, and soil data on the various experimental dates are provided in Table S1 in the supplemental material. Each batch of fresh *P. giganteum* was immediately cut into about 2-cm lengths with a feed cutter, mixed thoroughly, and split into two parts for silage preparation and fresh sample analysis, respectively.

### Silage preparation.

A total of 36 bags (2 treatments × 6 fermentation times × 3 replicates per treatment) were prepared, and the treatments were set as follows: (i) natural fermentation of P_A_ (NP_A_) and (ii) natural fermentation of P_B_ (NP_B_). Specifically, approximately 0.45 kg of mixed material was packed into a presterilized laboratory silo (polyethylene plastic bag with a size of 300 by 400 mm), sealed with an Aomitai DZD-400 vacuum sealer (Aomitai Technology Co., Ltd., Jiangsu, China), and kept under ambient temperature (28 ± 3°C) for 1, 3, 7, 15, 30, and 60 days of ensiling.

### Chemical and microbial composition analysis.

Before analyses, fresh or ensiled *P. giganteum* was blended thoroughly. About 300 g of fresh or ensiled *P. giganteum* was oven dried at 105°C for 15 min (deactivation of enzymes) and 65°C for 48 h to determine dry matter (DM). Subsequently, the oven-dried sample was ground by a laboratory knife mill equipped with a 1-mm screen. The water-soluble carbohydrate (WSC) content of fresh or ensiled *P. giganteum* was determined by the anthrone-sulfuric acid method (47). The neutral detergent fiber (NDF) content and acid detergent fiber (ADF) content of fresh *P. giganteum* were determined by using an Ankom 200 fiber analyzer (Ankom Technology, NY, USA). The total nitrogen (TN) content of fresh *P. giganteum* was determined by use of a Kjeltec 8200 Kjeldahl nitrogen analyzer (Foss Analytics, Höganäs, Sweden). The crude protein (CP) content of fresh *P. giganteum* was calculated by multiplying the TN content by 6.25 (48). The buffering capacity (BC) of fresh *P. giganteum* was determined by titration (49).

After extraction of 20 g of fresh or ensiled *P. giganteum* with 60 mL of deionized water at 4°C for 24 h, the extract was filtered with four layers of sterile gauze and Whatman filter paper. Then, the pH of fresh or ensiled *P. giganteum* was determined by using a Hanna HI 2221 pH meter (Hanna Instruments, Inc., RI, USA). The ammonia-nitrogen (NH_3_-N) concentration of ensiled *P. giganteum* was determined by the phenol-hypochlorite method (50). The concentration of lactic acid (LA), acetic acid (AA), propionic acid (PA), and butyric acid (BA) of ensiled *P. giganteum* was quantified by an Agilent 1260 Infinity high-performance liquid chromatography system (Agilent Technologies, Inc., Waldbronn, Germany) as reported in our previous work (51).

About 10 g of fresh or ensiled *P. giganteum* was homogenized with 90 mL of autoclaved 0.85% sodium chloride solution and shaken at 120 rpm, at 37°C, for 2 h. Subsequently, 1 mL of the above-described solution was serially diluted at a 10-fold gradient for microbial enumeration. The LAB, aerobic bacteria, yeasts, molds, and enterobacteria were counted by culture-based methods using selective media (52). Specifically, LAB were counted on de Man, Rogosa, and Sharp agar medium after 72 h of anaerobic incubation at 37°C. Aerobic bacteria were counted on nutrient agar medium after 48 h of aerobic incubation at 37°C. Yeasts and molds were counted on potato dextrose agar after 48 h of aerobic incubation at 30°C. Enterobacteria were counted on violet red bile glucose agar medium after 24 h of aerobic incubation at 37°C. The microbial number was enumerated in CFU, converted to logarithmic form, and expressed on a fresh material (FM) basis. After filtration with two layers of sterile gauze, the obtained filtrate was collected for subsequent bacterial DNA extraction.

### High-throughput sequencing analysis.

The bacterial DNA extraction and PCR amplification were conducted according to the procedures reported in our previous work (53). In brief, the solution used for bacterial DNA extraction was centrifuged at 10,000 × *g* for 15 min at 4°C to collect microbial precipitates. The bacterial DNA was extracted by using a FastPrep-24 instrument and a FastDNA spin kit (MP Biomedicals, CA, USA), and the quantity and quality of extracted DNA were determined by using 1% sodium borate agarose gel electrophoresis and a NanoDrop 2000 UV-visible spectrophotometer (260/280 nm; Thermo Scientific, DE, USA). According to the designated sequencing area, specific primers with barcodes were synthesized. The V3-V4 hypervariable region of bacterial 16S rRNA gene was amplified by use of an ABI GeneAmp 9700 PCR amplification instrument (Applied Biosystems, CA, USA). The amplification primers were 338F (5′-ACTCCTACGGGAGGCAGCAG-3′) and 806R (5′-GGACTACHVGGGTWTCTAAT-3′). Each sample was run in three PCRs to reduce PCR bias. The PCR products of the same sample were mixed and detected by 2% agarose gel electrophoresis. The PCR amplification products were recovered by use of an AxyPrep DNA gel extraction kit (Axygen Biosciences, CA, USA), eluted with Tris-HCl, and purified by 2% agarose gel electrophoresis. The purified amplicons were paired-end sequenced on the Illumina MiSeq PE300 platform (Illumina Inc., CA, USA). The FLASH software (v1.2.11) was selected to process all raw reads, and the QIIME software (v1.9.1) was used to filter out low-quality sequences with quality scores of less than 20. The UCHIME algorithm was applied to remove chimeric sequences, and the UPARSE pipeline (v7.0) was chosen to cluster the qualified reads with 97% identities into operational taxonomic units (OTUs). Based on the SILVA 132 database, the above-mentioned OTUs were classified and denominated at the phylum and genus levels using the RDP classifier (v2.11) with a confidence threshold of 70%. *Lactobacillus* genera were reclassified in 2020 and split into 25 new genera due to their extreme differences in ecology, phenotype, and genotype levels (54). Due to the limited taxonomic resolution allowed by 16S rRNA metabarcoding, the annotation of *Lactobacillus* has not been changed. The annotated *Lactobacillus* species in this study represented the sum of *Lactobacillus*, *Lacticaseibacillus*, *Lactiplantibacillus*, *Latilactobacillus*, *Liquorilactobacillus*, *Levilactobacillus*, *Lentilactobacillus*, *Loigolactobacillus*, *Limosilactobacillus*, *Fructilactobacillus*, *Companilactobacillus*, *Acetilactobacillus*, and *Apilactobacillus* based on the SILVA 138 database annotation. The QIIME software was also used to calculate bacterial alpha diversities, including Chao1, Shannon, and coverage indexes as well as beta diversities presented by the Bray-Curtis metric. The Vegan package of R software (v4.0.5) was loaded to construct principal-coordinate analysis (PCoA) plots for visualizing the Bray-Curtis distance metric. The pheatmap package of R software was loaded to construct Spearman correlation heatmaps for visualizing the linkages between chemical composition and phyllosphere microbiota (fresh samples) as well as fermentation parameters and silage microbiota (ensiled samples).

### Cooccurrence network analysis.

The cooccurrence pattern was constructed by calculating multiple abundance correlations based on a genus-level matrix using the Networkx toolkit (v2.6.3). Only genera with a relative abundance of >0.05% were considered. If the Spearman correlation coefficient (ρ) is >0.50 and the *P* value is <0.05, cooccurrence is considered to be robust. The cooccurrence networks were visualized using the interactive platform Gephi (v0.9.2). Nodes represent individual bacterial genera, and edges represent the pairwise correlation between nodes in the bacterial network. The calculated topological characteristics of bacterial networks include positive (cooccurrence) and negative (mutually exclusive) correlation numbers, network diameter, average shortest path length, average clustering coefficient, degree (average connectivity), closeness centrality (the average shortest distance from the given node to other nodes), betweenness centrality (the fraction of shortest paths passing through the given node), and modularity, etc. The keystone taxa were determined based on the combined score of low betweenness centrality, high closeness centrality, and high mean degree (34).

### KEGG functional prediction analysis.

The functional profiles of the bacterial community were predicted using the Tax4Fun tool. First, the 16S rRNA profile based on the SILVA database was transformed into a taxonomic profile of prokaryotes based on the KEGG database. Then, the 16S rRNA copy number was applied to normalize the abundance of KEGG prokaryotes. Finally, the functional profiles from high-throughput sequencing data were predicted by the linear combination of KEGG prokaryote functional profiles and normalized taxonomic abundances (11).

### Statistical analysis.

The effects of growth stage, fermentation time, and their interactions on fermentation characteristics, chemical composition, microbial number, and alpha diversity indexes of silages were investigated using the general linear model (GLM) of SAS (v9.2; SAS Institute, Inc., NC, USA) according to the model as follows:

$$Y_{\mathrm{ij}}=\text{μ}+G_{i}+T_{j}+{\left(G\times T\right)}_{\mathrm{ij}}+e_{\mathrm{ijk}}$$

where *Y_ij_* is the dependent variable, μ is the overall mean, *G_i_* is the effect of growth stage (*i* = 2, P_A_ versus P_B_), *T_j_* is the effect of fermentation time (*j *= 6, 1, 3, 7, 15, 30, and 60 days), (*G* × *T*)*_ij_* are the interaction effects of growth stage and fermentation time, and *e_ijk_* is the residual error. Comparisons between growth stages were analyzed using a *t* test when the effect of growth stage was significant. Meanwhile, the Student's *t* test and analysis of similarity (ANOSIM) with 999 permutations were adopted to statistically compare the differences in alpha diversity and beta diversity of the bacterial community, respectively. The statistical differences were considered significant at a *P* value of <0.05.

### Data availability.

The sequenced data were deposited in the NCBI Sequence Read Archive (SRA) database under BioProject accession no. PRJNA850133.
