# Supplementary material for: Dynamics of Phyllosphere Microbiota and Chemical Parameters at Various Growth Stages and Their Contribution to Anaerobic Fermentation of Pennisetum giganteum
Source: Microbiol Spectr. 2023 Apr 3;11(3):e02288-22. doi: 10.1128/spectrum.02288-22 (PMC10269755; doi:10.1128/spectrum.02288-22)
Supplement: Supplemental file 1 — Supplemental material. Download spectrum.02288-22-s0001.pdf, PDF file, 0.3 MB [file spectrum.02288-22-s0001.pdf]

Supplementary Information

Dynamics of phyllosphere microbiota and chemical parameters at various growth stages and their contribution to anaerobic fermentation of *Pennisetum giganteum*

Jie Zhao, Hao-Peng Liu, Xue-Jing Yin, Zhi-Hao Dong, Si-Ran Wang, Jun-Feng Li, Tao Shao*

Institute of Ensiling and Processing of Grass, College of Agro-grassland Science, Nanjing Agricultural University, Nanjing 210095, China

*Corresponding author: T. Shao. E-mail: taoshaolan@163.com

Content catalog

Table S1. The temperature, precipitation and soil data on the various experimental dates 2

Fig. S1. Scatter plots of chemical compositions and fermentation products 3

Fig. S2. **S**catter plots of microbial number 4

TABLE S1. The temperature, precipitation and soil data on the various experimental dates

Item and experimental date	May 20, 2020	August 16, 2020	September 26, 2020
Mean temperature (℃)	17.8	32.2	21.7
Maximum temperature (℃)	30.0	37.0	26.0
Minimum temperature (℃)	16.0	26.0	18.0
Precipitation (mm)	0.00	0.00	0.00
Soil type	Sandy loam	Sandy loam	Sandy loam

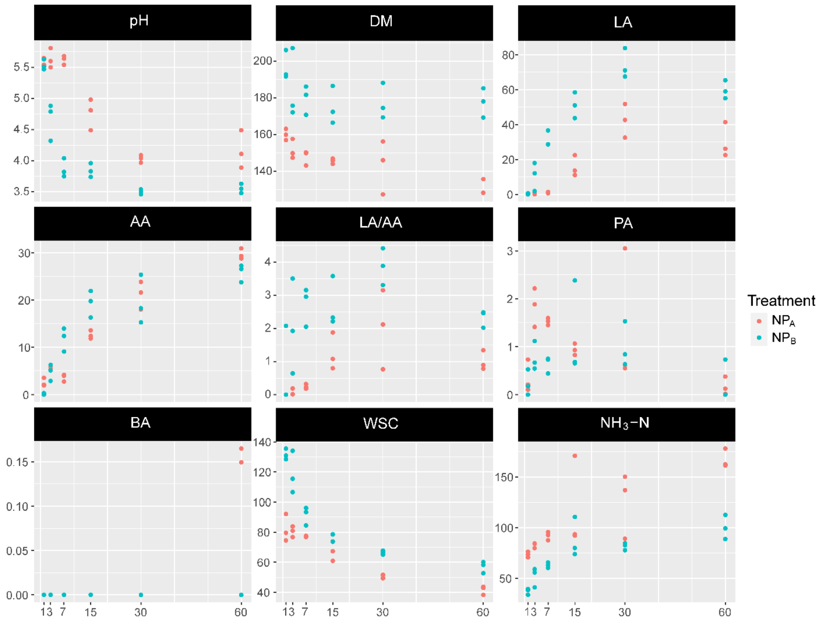

Fig. S1. Scatter plots of chemical compositions and fermentation products.

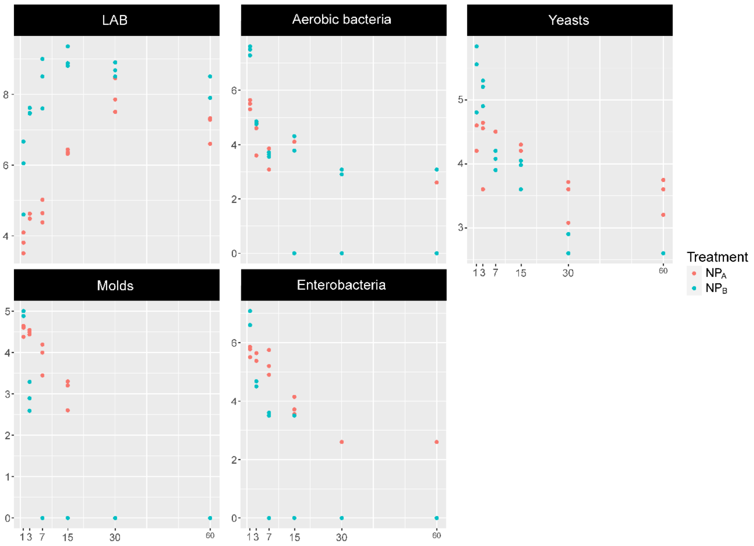

Fig. S2. Scatter plots of microbial number.
